# Periscope

**Published:** 1887-03

**Authors:** 


### PERISCOPE.

### PHYSIOLOGY.

**Sodium Chloride.**—What part does this play in the animal economy? It does not enter into the composition of any tissue, but all the physiological fluids contain it in notable proportions. The flesh of fish which live in sea-water has not a salt savour. Liebig believed that most of the sodium phosphate met with in animal bodies is formed from the potassium phosphate of plants, by double decomposition with sodium chloride. A solution of these salts, allowed to evaporate under a bell-glass covering, also a dish of conc. sulphuric acid, after some days deposited undoubted crystals of sodium phosphate. It is obvious that if common salt similarly favours the formation of this phosphate in a living organism, it is a physiological fact of great interest, and one which gives to this universal condiment a very important *rôle* with animal economy.—*Journ. de Med. de Paris*, and *Amer. Pract. and News*, Dec. 11th, 1886.

ARTHUR B. PROWSE, M.D.

### MEDICINE.

**Raynaud's Disease.**—The marked feature in these cases is the localised temporary blanching of the fingers, toes, nose, ears, &c., followed in some cases by localised gangrene. Dr. Shaw, in a paper read before the Brooklyn Patholog. Society, April 22nd, 1886, gives details, from various sources, of 10 cases. Two were men, aged 32 and 51 years; the remainder were females, aged respectively 5, 5, 9, 32, 40, 41, 50, and 51. In most of these patients there was either abnormal irritability of the nervous system, or positive nervous disorder. Cold, emotional disturbances, fright, &c., appear to be exciting causes. In many of the cases the affection is symmetrical. In some it is associated with intermittent hæmaturia, and in others with diabetes. In the *chronic* form, lasting some months, there may be localised gangrene, followed by cicatrization, as in the acute; but more commonly there are manifested other alterations in the nutrition of the parts, so that the skin becomes much thickened and indurated, resembling scleroderma. Sensation (?) and motion are said to be unimpaired ; and the temperature of the affected parts may be lowered or elevated. The cause of the condition is believed to be vaso-motor spasm. As to treatment: galvanism locally, and to the spine, has been very generally used. Potassium bromide and iodide, arsenic, quinine and other tonics, are variously recommended; and where there is pain, morphia internally, and chloroform or light bandaging locally. In cases complicated with diabetes, scleroderma, &c., the treatment indicated for those conditions. —*N. Y. Med. Journ.*, Dec. 18th, 1886.

**Three Attacks of Scarlatina.**—A second attack of scarlatina is a rare event. It has never been seen by such authorities as Hebra and Bouchut. Dr. N. Woronochin, of St. Petersburg (*Jahr. f. Kind.*, B. xxv., H. 1, 2, 1886), reports a case in which a written record of each of three attacks was preserved, and the observations may fairly be assumed to be positive and free from error. The first attack was in 1868, when the patient was three years old: there was only a slight rash, but free desquamation. In the second attack, at the age of 10, there was a well-marked rash, with sore throat and swollen glands, followed by desquamation. The third attack, at the age of 21, lasted longer than the others: the rash was very abundant, and the desquamation characteristic; the other symptoms being also well marked. The patient was suffering from gonorrhœa for a fortnight before this attack began, and was treated with copaiba. It was at first thought the rash might be due to the drug; it was, therefore, continued during the illness, and appeared to exercise no influence whatever on the course of the fever.—*Edin. Med. Journ.*, Jan., 1887.

[During nine months' residence at the London Fever Hospital, I saw, among several hundred scarlet-fever patients, two undoubted cases of second attacks. One was in a man, aged about 20, employed at a large dairy, who had been in the hospital eleven months previously with an attack of moderate severity. On the second occasion the signs were well marked, and the course of the affection characteristic. The second case was that of a man, aged 43, who, after a somewhat mild attack, peeled freely. One month after his admission, the throat again became sore, the cervical glands enlarged, a bright punctiform rash appeared, and the fever was high. A few days later rheumatism set in, followed by pericarditis, which soon proved fatal. A *p.m.* examination showed that the pericardial fluid was purulent.]

**Actinomycosis.**—At the Chicago Med. Society (Nov. 1st, 1886), Dr. A. J. Oschner showed a case of this disease. The patient, aged 56, had been engaged for more than forty years in cattle farming. Many of the animals suffered from "lumpy jaw," and it was his practice to cure them by free crucial incision, turning out the contents, and introducing about ʒi. of "powdered arsenic" into the cavity. In 1877 he began to suffer severe neuralgic pains in the left superior maxillary region; and six months afterwards an abscess opened into the pharynx, giving much relief. The antrum was then opened above the first molar tooth, and the curette applied. Irrigation was continued two or three times daily for two years. Since July, 1885, cough had been persistent, the expectoration being muco-purulent and streaked with blood. Microscopical examination showed the presence in it of the characteristic fungus. There were signs of partial consolidation in the left lung, above the fifth rib. Elsewhere the lungs appeared normal, and there was no evidence of disease in any other part of the body. The present treatment is inhalation of $\frac{1}{10}$ p.c. solution of corrosive sublimate.

A report of the pathological committee was read on another case of the same disease in a feeble, emaciated man, 25 years old, exhibited to the Society on Sept. 6th. "The lateral diameter of the neck is much increased by inflammatory thickening of the superficial tissues. On each side of the neck is a small fistulous opening, exuding a slight serous discharge. The jaws can only be separated to a slight extent; but, as far as can be determined, the mouth and throat present nothing abnormal. No carious teeth can be detected. There is evidence of consolidation in both apices: on the right side as low as the third rib, and on the left to the first intercostal space. There is frequent cough, and much expectoration. Specimens of the actinomycetes have been obtained from the sputum, and also from a small abscess in the neck."—*Medical Age*, Detroit, Dec. 10th, 1886.

ARTHUR B. PROWSE, M.D.

**Albuminuria in Syphilitic Patients.**—A report of the Paris Society of Medicine ends with the following conclusions:

(1) Syphilis in the first months of its appearance may give rise to albuminuria.

(2) This albuminuria is easily cured by anti-syphilitic treatment without leaving any ill effects, and must be distinguished from that which appears during the second or third year of the course of the disease. In the latter case the appearance of albuminuria is of very serious significance, as it is probably the starting point of a chronic nephritis.

(3) Syphilitic patients, on account of the malaise always produced by syphilis, become more susceptible to cold, and therefore more predisposed to nephritis arising from cold and exposure.

J. Michell Clarke, M.B.

### THERAPEUTICS.

**Goitre treated by Carbolic Acid.**—Dr. O. E. Haven states, in the *Weekly Med. Record* (Sept. 11th, 1886), that he has seen a large number of cases of goitre in the public clinics in Chicago, all of which have been materially benefited and almost all cured by the injection of 20 or 30 minims of 5 p.c. solution of carbolic acid, once or twice a week, into the substance of the gland. To do this thoroughly, the needle of the hypodermic syringe is inserted half to one inch into the gland: little or no pain is felt, but only a sense of dizziness, lasting a few minutes. Contraction and hardening ensues; and in eight or ten weeks the tumour has subsided. In only two cases out of 150 did slight inflammatory symptoms supervene, and these subsided in a day or two.—*Therapeutic Gazette*, Dec., 1886.

**Thymol as a Tæniafuge.**— Dr. Numa Campi (in *Il Racoglitore Medico*) recommends this drug strongly. It is very slightly soluble in water, but easily in alcohol, ether, or alkaline solutions. Its taste is warm, bitter, and acrid. As a germicide and antiseptic, it has been found to be second only to mercuric chloride. It has proved efficient in destroying the anchylostoma duodenalis, the parasite which gives rise to what is known as "miner's anæmia." The method is as follows: Half-an-ounce of castor oil is to be taken, fasting, in the evening; the next morning, ʒii. of thymol divided into twelve doses, one to be taken every quarter-hour; to be followed by a second dose of castor oil. The drug is rather depressing, so it is well to give some stimulant during its administration. Up to the present, Dr. Campi has not found it fail.—*Therapeutic Gazette*, Dec., 1886.

**Ergot for Headaches.**—Dr. Sturgis, in the *Boston Med. and Surg. Journal*, recommends ext. ergotæ liq., in doses of ♏ x. thrice daily after meals, as very effectual in some of the headaches of children. He does not, however, indicate very clearly the character of the headaches benefited thereby.

**Morphine in Diabetes.**—Dr. Mitchell Bruce, in the *Practitioner* (Jan., 1887), has a paper founded upon observations on a diabetic man, 26 years of age, who was under treatment ten months. The observations were arranged in four series. The first extended over two days only, while the patient was on ordinary mixed diet and no medicine. The daily excretion of glucose at this time was 1,360 grains. The second series related to the patient's condition while on "strict Pavy diet," and no medicine beyond aq. camph. During the twenty-three days this was persevered with, the average amount of sugar was the same—viz., 1,360 grains. In the third period of sixty-eight days the rigid diet was continued, and solution of acetate of morphia given by the mouth in doses, at first of ♏ xv. every four hours, increased every three or four days until the daily amount ingested was $4\frac{3}{4}$ grains of the solid drug. Very slight drowsiness was produced by this quantity. The amount of sugar excreted steadily decreased as the medicine was increased, until at length none could be detected. For seven days now the strict diet was continued, but the drug omitted; the result being that sugar reappeared in the urine, and quickly increased to 2,400 grains per diem. In the fourth period the dieting was maintained, and morphia was given hypodermically, commencing with gr. $\frac{1}{4}$ thrice daily. This was increased by $\frac{1}{12}$ gr. every three or four days until a maximum of grs. iii. per diem was reached, and then drowsiness necessitated a diminution of the amount. At no time during this period of forty-nine days was the sugar less than 768 grs. in the day. A fifth series of observations was subsequently commenced, when the average daily amount of sugar was 4,000 grs., on rigid diet, and no medicine. Morphia was given by mouth again, commencing with gr. $\frac{1}{4}$ every four hours. On the ninety-ninth day, when exactly 7 grs. of the acetate was being taken, the sugar was reduced to a mere trace.

The physiological and pathological deductions the author draws are the following: (*a*) The glycosuria (in this patient) was proved to be due to an increased income of sugar to the blood, not to a diminished destruction of the sugar in the system. This is evident, from the fact that the drug had much less influence when introduced into the general circulation—where destruction of sugar is accomplished—than when introduced into the portal circulation. (*b*) The increased income of sugar was proved not to originate in simple transportation of sugar from the intestine or portal vein to the general circulation; for when all saccharine and amylaceous materials were excluded from the diet, the patient continued to excrete large quantities of sugar. It was clear, therefore, that glycogenesis was still active, and that the sugar was derived from the nitrogenous constituents of the food. (*c*) The excessive glycogenesis that was going on was proved to be effected mainly or entirely in the liver, not in the muscles or any of the other viscera. This followed from two facts: (1) That when the morphia was introduced into the liver by the portal vein it reduced the sugar to *nil*, whilst it did not materially affect the other viscera, such as the central nervous system; and (2) that when it was introduced into the general circulation it diminished the amount of sugar excreted only to the degree that might have been anticipated from the portion that would reach the liver through the hepatic artery, whilst it markedly affected the other viscera, as shown by the drowsiness. (*d*) "These results appear to prove that in this instance, if the diabetes was of nervous origin, the seat of the disordered process—I do not say the seat of the nervous derangement originating it—was in the liver, not in the central nervous system or nerve-trunks." This also followed from the effect of morphia by mouth, as compared with its effect *sub cute;* for by the latter method the central nervous system was profoundly depressed, while there was but little effect on the glycosuria—through this channel probably *none*.

**Antipyrin in Rheumatism.**—Dr. Eich, in his inaugural essay (Basel, 1886) on the anti-rheumatic virtues of this drug, concludes that it is very prompt and reliable in its action, and is in no way inferior to the salicylates. In a very few cases it appears to fail just as the salicylates do. No unpleasant secondary effects were noticed. The daily dose was 60 to 90 grains at the outset, diminished later on to 30 grains.—*Therapeutic Gazette*, Nov., 1886.

M. Clément, of Lyons, from his experience in the Hôtel Dieu, prefers antipyrin to sodæ salicylas in the treatment of acute and subacute rheumatism. No vertigo, deafness, or tinnitus follows its use; and he denies that it is a cardiac depressant. In two cases, complicated with pleurisy and pericarditis, there was such a rapid absorption of the serous effusion that he is inclined to think it was materially assisted by the drug. He has always continued the administration for some time after the pain and fever have gone, and has found it easily tolerated even by weak stomachs. A daily dose of 60 to 90 grains was found rapidly effectual. In chronic rheumatism he found it of doubtful value, and in gonorrhœal pseudo-rheumatism it was of no more use than the salicylates. In all cases in which it failed sod. salicyl. failed too; and in a few cases in which the latter failed it succeeded.—*Lyons Médical*, Aug. 29th, 1886, and *Practitioner*, Jan., 1887.

In the *N. Y. Med. Journ.*, Oct. 24th, 1886, Dr. Eccles states, that while making pharmaceutical investigations, he had occasion to mix some spt. eth. nitrosi with a solution of antipyrin. The mixture was at first clear and colourless, but after some hours became green. The optical appearances of the fluid were identical with those of the *anilines;* and as antipyrin was a coal-tar product, he supposed the change was due to the formation of a green aniline; and so it proved to be. This incompatibility is important, because both substances are used in febrile states, and might happen to be prescribed together.—*Med. Age*, Detroit, Dec. 10th, 1886.

**Sodium Chloride in Bright's Disease.**—Dr. Meminger, of Charleston, recommends this in consequence of the good results obtained in four chronic cases. Gelatine capsules containing 10 grains of common salt are given thrice daily, and on alternate days before and after meals. The number is increased every day until five capsules are being taken *ter die*, when improvement begins to be manifest, both in the state of the urine and in the general condition of the patient. After this, six capsules *per diem* are usually sufficient. Should nausea supervene, the recumbent posture, or a temporary omission of the drug is advised, and one or two alterative pills should be given.—*N. Y. Med. Journ.*, July 31st, 1886.

**Trichlorophenol** is prepared from carbolic acid by substituting three atoms of chlorine for three of hydrogen, thus: $C_6H_6O + Cl_3 = C_6H_3Cl_3O + H_3$. It is said to be a much more powerful antiseptic than phenol, and is being tried extensively both in Russia and the United States.—*Amer. Pract. and News*, Dec. 11th, 1886.

**Lanolin Salves.**—Dr. P. Bauskinski has made a series of test experiments, in regard to the absorption of various substances in lanolin salves, which antagonize the assertions of Liebrich and Lassar. He concludes that lanolin possesses no exceptional advantages over other fats, except its neutral reaction and slight destructibility; still, it is in most cases a good material for salves. "It is doubtful, as yet, whether we can be sure of the impossibility of communicating splenic fever by its use."—*Deutsche Med.-Zeitung*, and *Amer. Pract. and News*, Dec 11th, 1886.

**Drumine** is a new local anæsthetic obtained from Euphorbium Drummondii by Dr. J. Reid, of Port Germain,

South Australia. Cocaine has a mixed sensory and motor action, and causes preliminary excitement; but Drumine appears to be a purely sensory paralyser, and does not stimulate primarily. It does not affect the pupil. Given subcutaneously it was very successful (in doses of ♏ iv. of a 4 p.c. solution) in removing sciatica and the pain of sprains; and when dropped into the eye, or applied to the skin locally, was effectual in "tic."—*Australasian Med. Gazette*, Oct. 15th, 1886.

**Quillaja Saponaria.**—A $2\frac{1}{2}$ p.c. decoction of this bark is a valuable expectorant, in conditions similar to those for which senega is prescribed, in doses of ℥ ss. for adults, or ʒi. for children. Dr. Kobert states that it contains the two glucosides found in senega, but in five times greater amount, whilst its cost is only $\frac{1}{10}$th that of senega. A common property of the two drugs is that, in consequence of the presence of *saponine*, 5 or 6 drops of the tincture will emulsify ℥ ss. of fixed oil. This property may be utilised in ordering turpentine, terebene, or eucalyptus oil, in cough mixtures. Quillaja contains enough sugar to sweeten its decoction, which is altogether more palatable than decoct. senegæ. Goldschmidt (*Aertz. Intelligenzbl.*, 1885) has confirmed Kobert's conclusions.—*Edinburgh Med. Journ.*, Jan., 1887.

ARTHUR B. PROWSE, M.D.

**Diet in its relation to Therapeutics.**—Under this title M. Dujardin-Beaumetz considers, the normal physiological diet being known, what is the effect of reducing or increasing the amount of this diet, and in what ways should the diet be modified in various maladies.

A smaller quantity of food is chiefly indicated in obesity, where it exists apart from acute disease or organic lesion. These patients are recommended to drink only white wine (clarets, hocks), and these diluted with an alkaline water in a quantity not exceeding 9 oz. at each meal; to eat only the crust of bread and no pastry; never to take tea with sugar between meals, sweet wines, beer, or liqueurs; to use purgatives frequently; to take much exercise and undergo massage.

Superalimentation finds its chief use in phthisis in the form of meat powders.

The author considers that the diet of the gouty, and of those who suffer from increased production or deficient elimination of uric acid, should be a mixed one; total abstinence from purely nitrogenous food, so long recommended, being a mistake. As regards meats, all kinds may be used, preference being given to the white kinds; eggs, fish, shell-fish, "ripe"

cheeses, and fats, to be taken sparingly. Vegetables should form a large portion of the diet, except those highly azotised, such as cabbage, broccoli, peas, beans, lentils, mushrooms, and truffles: these latter may, however, be used in smaller quantities. Spinach and sorrel only are forbidden. Fruits are recommended, especially grapes and strawberries. These patients should be encouraged to drink abundance of liquids, on account of their diuretic effect—for preference, water or alkaline waters; old wines containing little tannin, light wines containing little alcohol mixed with some alkaline water, and weak coffee, are admissible in moderate quantity. Champagne, beers, porter, artificial aerated waters, tea, liqueurs, sweet and "fruity" wines, are absolutely prohibited. The meals to be moderate in quantity, sufficient for nutrition without overloading the stomach, and regular.

In oxaluria, any food that tends to the formation of oxalic acid is to be avoided—*e.g.* highly spiced food and condiments, tea, cocoa, coffee, chicory, chocolate; peas, beans, celery, cauliflower, spinach, rhubarb, endive; dried fruits, oranges and raspberries.

In hepatic gravel, all fats, carbohydrates, sugar, and feculents that give rise to the formation of cholesterine, the determining cause of this gravel, are forbidden. Peas and carrots, that contain a body closely allied to cholesterine, are especially injurious. In short, the diet in these cases is to consist of lean meat and green vegetables, of fruits that have little sugar, of little bread, of no feculent foods, except potatoes, and of no pastry. If any wine is taken, it is to be diluted with alkaline waters.

In diabetes, M. Dujardin-Beaumetz considers potatoes especially valuable, inasmuch as they contain the least starch of all feculents, and recommends them in preference to gluten bread, which must be used instead of ordinary bread. All kinds of meat and fish, soups, clear or thickened with vegetables, except peas or beans, are allowed. Foods containing much fat, and vegetables, except carrots, parsnips, and beetroot, are recommended—more especially vegetables, from the variation they make in the dietary. All feculents, except potatoes, all fruits, alcoholic drinks, except wine mixed with a natural alkaline water, are forbidden. The patient should not drink anything between meals, unless he suffers from great thirst, when weak tea, or coffee without sugar or milk, or weak infusions of quassia or cinchona, are permissible. Muscular exercise and massage are useful as aiding the elimination of sugar.

In chronic albuminuria, milk takes the first place, with the addition of fruits, vegetables, feculents, and fats. Eggs and meat of all kinds are injurious. If the appetite fails under the above *régime*, pork, especially fat pork, ham, or bacon, are preferable to other forms of meat. Milk is the best drink; but if the patient cannot take it for a long time, a light wine containing much tannic acid, mixed with an alkaline water, may be permitted. Since it has been proved that the quantity of albumen in the urine is increased after large meals, the meals should be small and often.

As to the use of alcohol in fever, M. Dujardin-Beaumetz considers that it is a true food in this condition, and that its action lies in undergoing more or less complete combustion in the organism at the expense of the oxygen of the blood, thereby diminishing the destructive metabolic process taking place in the tissues and lowering the temperature, or, in other words, checking tissue waste. At the same time, it gives strength and tone to the nervous system.—*Gazette des hôpitaux*, Nov. 20th and 27th, 1886.

**Treatment of Diphtheria.**—M. Brondel, practising at Algiers, states that by the following mode of treatment he has not lost a single case of diphtheria out of a total number of 200 treated in the last five years:

The method consists in the employment of benzoate of soda, vaunted by Letzerich as a true specific in diphtheria, in doses of 6—7½ grains in solution, according to the child's age, together with gr. $\frac{1}{7}$ sulphide of calcium, in pill or in syrup, every hour from the onset of the disease. A 10 °/₀ solution of benzoate of soda is sprayed into the throat by an atomiser every half-hour, regularly throughout the day and night in bad cases, but in those less severe the patient is not disturbed during sleep.

The above treatment must be rigorously carried out. The spray does not injure the throat in the slightest, if properly managed: under its influence the false membranes become pale, less consistent, more and more gelatinous, and finally disappear, dissolved by the benzoate of soda, leaving the underlying surface healed. Continuous vaporisation of terebene, eucalyptol, or carbolic acid is carried out at the same time. [This can be done by charging the air of the room with moisture from a steam-kettle containing one of the above antiseptics.]

The food consists of meat-juices, eggs, and underdone meat, if the state of the throat permits. Arseniate of strychnine is recommended as a tonic; antipyrin, or quinine, to control any excessive temperature, if present. Free ventilation is to be ensured, and all linen and excreta thoroughly disinfected.

After the termination of the disease, the room is disinfected by sulphur.—*Gazette des hôpitaux*, Dec. 11th.

[Benzoate of soda has been given in doses of 380 grains daily without ill effect.]

**Treatment of Enteric Fever.**—Professor Bouchard has formulated new rules for the treatment of typhoid fever, based on his recent researches into the pathogeny of the disease. The chief indications are four: To render the tissues generally antiseptic, to disinfect the intestine, to combat hyperpyrexia, and to give nourishment.

When enteric is suspected, or has been diagnosed, M. Bouchard prescribes:

(1) A purgative administered every third day, and consisting of 3½ drachms of sulphate of magnesia.

(2) Six grains of calomel given daily in 20 divided doses, one dose every hour, for four following days, to produce general antisepsis.

(3) Disinfection of the intestine is carried out by a mixture consisting of—

| | |
|---|---|
| Powdered vegetable charcoal ... | 3½ ounces |
| Iodoform ... ... ... ... ... ... ... | 15½ grains |
| Naphthaline ... ... ... ... ... ... | 77 " |
| Glycerine ... ... ... ... ... ... ... | 7 ounces |
| Peptone ... ... ... ... ... ... | 1¾ " |

The above quantity to be taken in twenty-four hours, in doses of a tablespoonful every two hours in water.

He washes out the large intestine regularly night and morning with an injection of nearly a pint of water, containing 1 pt. carbolic acid in 1,000 pts. water.

(4) From the first day of the disease to complete convalescence the patient takes eight baths daily. The temperature of the baths is to be 2° C. below the temperature of the patient, very gradually cooled down to 30° C., never below that point, in order to obtain loss of heat without nervous shock or spasm of the peripheral vessels.

(5) Quinine is administered in the few cases in which the baths do not keep down the temperature. The indication for quinine is a rectal temp. of 104° F. in the morning, or 105.5° F. at night.

(6) The food consists of 50–70 oz. daily of broth made from meat and barley, and of the glycerine and peptone in the above mixture; lemonade is allowed with a little wine.

In prolonged and excessive delirium, opium is given.

The mortality from enteric in M. Bouchard's wards used to be 25%. Since April 1st, 1884, he has treated 266 patients on the new plan given above, and has lost 31, giving a mortality of 11% only.

The mean duration of the disease has been 19 days. Relapses, which used to occur in 20% of cases treated by the old methods, are now reduced to 10°/₀.—*Gazette des hôpitaux*, Jan. 22nd, 1887.

[Murchison gives the average mortality at the London Fever Hospitals as 17·5°/₀.]

J. MICHELL CLARKE, M.B.

**Acetanilide (or Antifebrin).**— This is a substance produced by the action of heat on aniline acetate. It has been found by Drs. Cahn and Hepp to be a powerful febrifuge in doses of 4 to 15 grains. Its action was not so evanescent as is often the case with antipyretics. Redness of the skin with moderate perspiration, thirst, and increased secretion of urine, were noticed in the twenty-four cases in which it was used. The skin of the extremities in some cases had, temporarily, a dark tint, but no unpleasant symptom was observed. It is an odourless powder, slightly soluble in water, readily in spirit. The taste is not unpleasant. The present price is one shilling an ounce.

**Peritoneoclysis, Hypodermoclysis, and Vesicoclysis in Cholera.**—A paper with the above title was read before the Bombay "Medical and Physical Society," on Feb. 6th, 1885, by Brigade-Surgeon C. Macdowall. In the *Lancet* of Oct. 13th, 1883, the author had recommended the use of large injections of warm water (plain or medicated), or of milk, &c., either subcutaneously or into the peritoneum, as a perfectly easy and safe means of reviving the circulation and the secretions, and prolonging life, in the collapse of cholera. He was then unaware that Dr. W. B. Richardson had tried the intra-peritoneal method in one case, in 1854, with success; and had also experimented (though not in cholera cases) with subcutaneous and intra-vesical injections. Absorption by the last method was found to be unsatisfactory. Intra-peritoneal injections of defibrinated blood have been employed successfully, in France, Italy, and Germany, as a remedy in pernicious anæmia. Hypodermoclysis with large quantities of water (not more than one-fifth of the body weight—say, about 20 pints) has been used with success in Italy in cholera. One or two grains of common salt must be added to each ounce of warm water: alkaline solutions are injurious.

The apparatus is extremely simple: a funnel, or a common large syringe without the piston, a piece of indiarubber tubing, and a medium-sized aspirator needle, is all that is necessary for subcutaneous clysis; while for peritoneoclysis, the sharp needle is replaced by a blunt cannula with an 'eye' at the side, as in a catheter. This is pushed into the abdominal cavity at the bottom of a small incision in the *linea alba.* It is an infinitely simpler and more practicable plan than intravenous injection. The author considers that hypodermoclysis is preferable to peritoneoclysis in most cases; though the latter should be resorted to if the former fails to produce good results. The order of efficiency he assigns is the following: (1) defibrinated blood; (2) milk, or solution of "white of egg;" (3) saline warm water.

ARTHUR B. PROWSE, M.D.

### DERMATOLOGY.

**Erythrasma and Eczema Marginatum.**—Von Bärensprung designated by the term erythrasma an affection of the skin presenting a yellow or brownish-red appearance composed of elevated circles or patches having a curved well defined outline, the surface of which is rough and scaly, and found almost exclusively in those localities of the body where two skin surfaces are in immediate contact. In reference to its pathogenic and clinical signification, we are compelled to express the opinion that erythrasma starts as a simple eczema intertrigo, upon which settle secondary fungi, adding to the primary disorder the characters of a parasitic lesion in its further course. If the Microsporon minutissimum, found upon the normal skin, comes in contact with the lesion, then the disease will take on the symptoms of erythrasma; but if the Trichophyton tonsurans be transmitted, the disease will then appear as an eczema marginatum, as described by Hebra. Chrysarobin, or Goa-powder, used in an ointment (10 p.c.), rapidly effects a cure.—Dr. G. BEHREND, of Berlin, *Med. Press of Western New York*, Dec., 1886.

**Dyed Hosiery and Skin irritation.**—The German Government, after a searching investigation, have permitted the use of all the aniline dyes, except picric acid, for dyeing goods and colouring even articles of food. Manufacturers of aniline dyes agree that they do not know of any skin irritations caused by anilines among the workmen, whose hands, &c., are constantly exposed to their influence. In these cases the dyes are in a soluble form; but in hosiery the colours are in as insoluble form as the dyer can ensure, and are therefore even less likely to be a source of trouble.—Mr. J. R. Ashwell, *Journ. Soc. Chem. Industry*.

Arthur B. Prowse, M.D.

### LARYNGOLOGY.

**Laryngeal Œdema.**—In the *N.Y. Med. Journ.*, Dec. 18th, 1886, is a paper read by Dr. De Blois, of Boston, before the American Laryngological Association, giving details of the fourteen cases of laryngeal œdema which he had met with, among 4000 throat cases, during a period of six years. "Occurring as they did among different classes of people, at different times of the year, and in different institutions, it seems to me that these cases form a very fair standard of the frequency of the affection, at least in this part of the world—that is, one in every three hundred throat cases." "The 245, then, that Sestier reports, would represent over 70,000 patients at the same ratio. Œdema of the larynx would appear to be more prevalent abroad than here." Charazac found that of 21 cases of acute primary laryngitis with œdema, 8 died; and of the remainder, some recovered after, and others without, tracheotomy. This mortality is very much less than in earlier times, for Sestier believed that most cases proved fatal. Of the 14 cases reported in this paper, 8 recovered after scarification, and 6 under treatment by astringents.

Arthur B. Prowse, M.D.

### PATHOLOGY.

**Mobility of the Heart.**—Dr. Shershevski has, in *Vrach*, a paper on this question. He gives details of the examination of 40 persons, all of them free from cardiac and pulmonary affections, in whom he noted accurately the position of the heart's boundaries in the upright, dorsal, and right and left lateral postures. The chief mobility was towards the left; but there was often very perceptible displacement to the right, as well as backwards, and even downwards. Youth, nervous states, and freedom of the vessels from signs of sclerosis, were favouring conditions. Backward displacement was found in nearly half the cases; and this suggests the wisdom of examining the heart in the upright posture, especially when results have to be compared. In subjects over 60 years of age, or in whom there was early signs of arterial degeneration, there was little or no mobility.

Arthur B. Prowse, M.D.

### STATE MEDICINE.

**A Travelling Hospital.**—The (1886) November number of the *Spitalul*, of Bucharest, contains, among other articles which show that our Roumanian brethren are intent on keeping pace with the latest advances in medicine and surgery, an interesting report by Dr. Bejan, on an itinerant dispensary and hospital system which is being applied in some of the rural districts of Roumania. Doctors are scarce outside the great towns of that country, and there is evidently a great field for philanthropic medical labour among the peasantry. Dr. Bejan's medical mission was organised on a military basis; and its aims were not limited to affording medical and surgical assistance to the rural population, but included hygienic and sanitary instruction, the collection of statistics of sickness, and the clearing of the ground, so to speak, for the speedy formation, whenever and wherever necessary, of an organisation to combat cholera or any other formidable epidemic.

Dr. Bejan's hospital and dispensary travelled, during the three months of summer, in the districts of Botosani and Dorohoï, succouring during that time upwards of 9,000 patients. Of these, about 7·5 per cent. were cases of malarial fever—not a very large proportion, considering the nature of the localities; but the proportion of cases of pellagra was not less than 23 per cent! and in Dorohoï the ratio was still larger. It would appear, therefore, that Roumania, whose climate might perhaps have been thought more propitious to the maize-plant than that of Upper Lombardy, is nevertheless equally the home of pellagra.

JOHN BEDDOE, M.D.

